# Low-carbohydrate diet in type 2 diabetes: stable improvement of bodyweight and glycemic control during 44 months follow-up

**DOI:** 10.1186/1743-7075-5-14

**Published:** 2008-05-22

**Authors:** Jörgen V Nielsen, Eva A Joensson

**Affiliations:** 1 Department of Medicine, Blekingesjukhuset, Karlshamn, 37480 Karlshamn, Sweden

## Abstract

**Background:**

Low-carbohydrate diets, due to their potent antihyperglycemic effect, are an intuitively attractive approach to the management of obese patients with type 2 diabetes. We previously reported that a 20% carbohydrate diet was significantly superior to a 55–60% carbohydrate diet with regard to bodyweight and glycemic control in 2 groups of obese diabetes patients observed closely over 6 months (intervention group, n = 16; controls, n = 15) and we reported maintenance of these gains after 22 months. The present study documents the degree to which these changes were preserved in the low-carbohydrate group after 44 months observation time, without close follow-up. In addition, we assessed the performance of the two thirds of control patients from the high-carbohydrate diet group that had changed to a low-carbohydrate diet after the initial 6 month observation period. We report cardiovascular outcome for the low-carbohydrate group as well as the control patients who did not change to a low-carbohydrate diet.

**Method:**

Retrospective follow-up of previously studied subjects on a low carbohydrate diet.

**Results:**

The mean bodyweight at the start of the initial study was 100.6 ± 14.7 kg. At six months it was 89.2 ± 14.3 kg. From 6 to 22 months, mean bodyweight had increased by 2.7 ± 4.2 kg to an average of 92.0 ± 14.0 kg. At 44 months average weight has increased from baseline g to 93.1 ± 14.5 kg. Of the sixteen patients, five have retained or reduced bodyweight since the 22 month point and all but one have lower weight at 44 months than at start. The initial mean HbA1c was 8.0 ± 1.5%. After 6, 12 and 22 months, HbA1c was 6.1 ± 1.0%, 7.0 ± 1.3% and 6.9 ± 1.1% respectively. After 44 months mean HbA1c is 6.8 ± 1.3%.

Of the 23 patients who have used a low-carbohydrate diet and for whom we have long-term data, two have suffered a cardiovascular event while four of the six controls who never changed diet have suffered several cardiovascular events.

**Conclusion:**

Advice to obese patients with type 2 diabetes to follow a 20% carbohydrate diet with some caloric restriction has lasting effects on bodyweight and glycemic control.

## Background

Type 2 diabetes reflects a disturbance in the glucose-insulin axis of metabolism and has insulin resistance as a defining feature. As such, it is expected that carbohydrate restriction would be the first line of attack and, in one form or another, this was the primary approach before the discovery of insulin [1]. In addition, at least anecdotally, some degree of carbohydrate reduction is a component of much clinical treatment. Health agencies have generally been reluctant to recommend carbohydrate restriction although the recent American Diabetes Association guidelines recognize that such diets are at least as effective as low fat diets for weight loss [2] and, while not recommending low carbohydrate diets, recognizes that dietary carbohydrate is the major factor in controlling blood glucose. Short term studies [3-7] in fact, demonstrate dramatic improvements in glycemic control even in the absence of weight loss [4].

Experience in our diabetes school showed that advice to reduce fat and increase carbohydrates had a very limited effect on long-term weight reduction in our obese diabetes patients (unpublished data). We therefore decided to test a different approach in an observational study with a control group. We were interested in seeing the effect of the diet in compliant patients, who could be expected to adhere to it 3–6 months. This enabled us to measure the actual effect of a carbohydrate-restricted diet with little contamination of non-compliant subjects. To this end, all patients were well-informed of the diet and its rationale before they started. We considered a weight reduction of 10% of bodyweight to be of clinical significance.

We have previously reported the results of these dietary changes over 6 months in 16 obese type 2 diabetes patients with a control group. In 2003, the 16 were advised to lower their carbohydrate intake to 20% of energy. In the course of 6 months, they achieved significantly better control of hyperglycemia and bodyweight than a control group of similar patients (n = 15) advised to follow the official guidelines where 55% carbohydrates is recommended [5]. We have further reported that the improvements were stable over 22 months [6].

We have now reviewed the clinical charts after 44 months and present the data for the intervention group with regard to glycemic control (HbA1c), bodyweight. Body mass index (BMI = weight/m^2^) and lipids after 44 months. We also report the results for 7 patients from the controls who immediately switched to a 20% carbohydrate diet and for whom long-term data is available.

## Methods

The method has previously been described in detail [5,6]. In short, the 16 patients, all with BMI>30 kg/m^2^, free of thyroid cardiac and renal disease – were advised to follow a diet containing initially 1800 kcal for men and 1600 kcal for women. The proportions of carbohydrates, fat and protein were 20%, 50% and 30% respectively. The daily quantity of carbohydrates was 80–90 g. The recommended carbohydrate consumption was limited to vegetables and salad. Instead of ordinary bread crisp/hard bread was recommended, each slice containing 3.5 to 8 g carbohydrates.

Excluded were starch-rich bread, pasta, potatoes, rice and breakfast cereals. The patients were counselled not to eat between meals. It was further recommended that they walk 30 minutes a day and take a daily multivitamin supplement containing extra calcium. There was an introductory meeting lasting most of one day. From day one diabetic medication was reduced by 25–30% to avoid hypoglycaemia. The patients monitored their own blood glucose 4 times a day and were counselled by telephone over the first few weeks for further reductions of medications.

The subjects were followed closely for 6 months with group follow-ups every second week for the first 3 months and once a months for the next 3 months.

The 15 controls were advised on a diet with about the same caloric content at an introductory meeting., Proportions of carbohydrates, fat and protein for this group were 55–60%, 25–30% and 15% respectively. In the normal diabetes diet whole-grain products are recommended. Generous helpings of vegetables and several servings of fruits as snacks between meals are also recommended.

As a number of the controls attended our normal diabetes educational course as introduction to the observation period, the control group on average received about 50% more attention – measured in hours – than the low-carbohydrate group. The controls were then followed in the same way as the low-carbohydrate group.

Seven of the 15 controls switched to a 20% carbohydrate diet immediately after the 6 months follow-up period. For those we have data 32–34 months after the change.

Three more controls sought information and attempted to change diet later at various dates. The 5 remaining controls have not attempted a change of diet despite receiving additional information.

All the patients were known to us and visited the diabetes nurse regularly after the initial period. The same scales and laboratory were used for all measurements. The present report is a review of clinical charts at about 44 months after the start of the study in 2003. Where a figure is missing at 44 months we have taken the mean from the two closest figures. Means are given with standard deviations. T-test for dependent samples is used.

## Results

Table 1 shows the measured parameters from start to 44 months. Triglycerides (TG) and HbA1c, both parameters of adherence to the diet, were at their lowest after 3 months. The adherence in the group then became less pronounced, again reflected in triglycerides and HbA1c.

**Table 1:** Effect of diet on weight, BMI, HbA1c and fasting lipids. Sixteen obese patients with type 2 diabetes started at month 0 on a diet with the proportions: 20% carbohydrates, 30% protein and 50% fat. The figures shown are means before, 3, 6, 22 and 44 months after the dietary change.

**Month**	**0**	**3**	P*	**6**	P*	**22**	**44**	P*
**Weight **(kg)	**100.6 **± 14.7	**91.9 **± 14.7	<0.001	**89.2 **± 14.3	<0.001	**92.0 **± 14.0	**93.1 **± 14.5	<0.001
**BMI **(kg/m^2^)	**36.1 **± 4.2	**33.0 **± 4.5	<0.001	**32.0 **± 4.3	<0.001	**32.9 **± 3.5	**33.4 **± 3.9	<0.001
**HbA1c **(%)	**8.0 **± 1.5	**5.9 **± 0.7	<0.001	**6.6 **± 1.0	<0.001	**6.9 **± 1.1	**6.8 **± 1.3	<0.001
**Lipids**(mmol/l)								
**Tot-Chol.**	**5.6 **± 1.2	**5.8 **± 1.1	0.4	**6.1 **± 1.1	0.06	**5.7 **± 1.2	**5.4 **± 1.0	0.8
**HDL-Chol.**	**1.1 **± 0.2	**1.2 **± 0.2	<0.002	**1.3 **± 0.2	<0.001	**1.3 **± 0.3	**1.3 **± 0.2	<0.001
§**Triglycerides**	**1.4 **(1;1.8)	**1.2**(0.8;1,4)	0.01	**1.4**(0.9;1.7)	0.4	**1.4**(1.2;1.9)	**1.4**(1.2–2)	0.9
**Chol/HDL**	**5.4 **± 1.5	**5.0 **± 1.5	0.02	**5.0 **± 1.7	0.07	**4.6 **± 1.6	**4.1 **± 0.9	<0.001
§**TG/HDL**	**1.4**(0.9;1.7)	**1.0**(0.6;1.2)	0.003	**1.0**(0.7;1.5)	0.03	**1.3**(0.8;1.5)	**1.1**(0.9;1.7)	0.7

§given as medians with 25 and 75 percentiles. * p values are for differences from baseline.

### Bodyweight

The mean reduction in bodyweight over the first six months was 11.3 ± 4 kg (controls: 1.8 ± 3.8 kg). Ten patients (62%) but none of the controls lost more than 10% of bodyweight.

**Figure 1:**
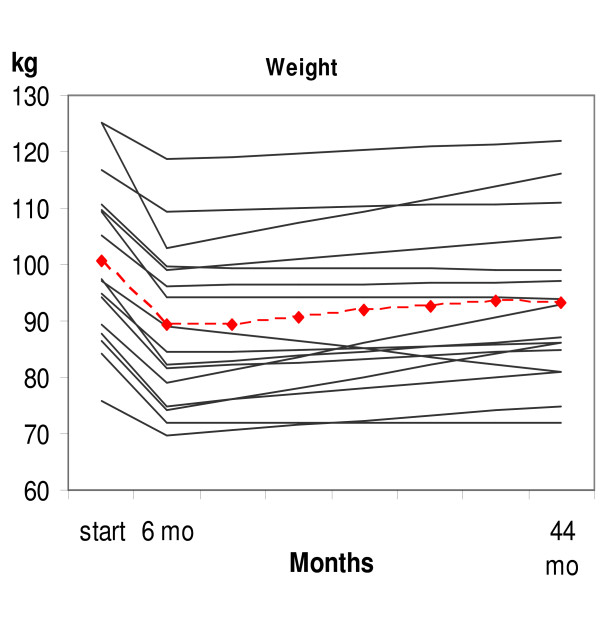
**Individual changes in bodyweight in 16 obese patients with type 2 diabetes**. The patients at start changed from a high-carbohydrate diet to a diet consisting of 20% carbohydrates, 30% protein and 50% fat. The dotted red line is the mean weight.

Mean bodyweight increased from 6 to 22 months by 2.7 ± 4.3 kg. The total mean increase from month 6 to 44 has been 3.9 ± 5.6 kg. Five of the patients have maintained bodyweight from 6 to 44 months or reduced it further (see figure 1). However, five patients have increased mean bodyweight by 10 kg. In 7 patients (43%) the bodyweight is still 10% or more below their original weight.

### HbA1c

The initial mean HbA1c in 2003 in the low-carbohydrate group was 8.0 ± 1.5% (controls: 7.9 ± 1.5%). At the end of the 6 months study period it was 6.6 ± 1.0% (controls: 7.3 ± 1.8%), and after 12 months it was 7.0 ± 1.3%. It has since remained stable and is 6.8 ± 1.3% after 44 months.

The effect of carbohydrate lowering on blood glucose was rapid. In the first week, mean fasting blood glucose dropped from 11.7 ± 3.3 mmol/l to 7.0 ± 1.4 mmol/l which necessitated corresponding reductions in medications.

### Medications

An important feature of carbohydrate restriction is that reduction and even elimination of antidiabetic medication normally is required in order to avoid hypoglycemia. At the start of the study 15 of the 16 used metformin and 5 sulfonylurea (SU). Eleven of the patients were treated with insulin at a mean daily dosage of 60 ± 33 IU.

All patients with SU reduced or discontinued it. Three patients of 11 discontinued insulin and the average insulin requirement of the last 8 persons was 18 ± 11 IU/day after 6 months.

After 22 months 2 patients had resumed insulin treatment following an increase of carbohydrates. The mean insulin requirement of the 10 persons after 22 months was 27 ± 21 IU/day. After 44 months the same 10 persons are still using insulin. The mean requirement is 41 ± 34 IU/day.

### The cross-over of the 7 controls

After the initial 6 months study period 7 of the 15 controls switched to a 20% carbohydrate diet. They had been followed closely during the 6 months on a low-fat, high-carbohydrate diet, and then – separated by a gap of 2 months-followed again with regular meetings for 6 months on a 20% carbohydrate diet. During this latter period, they achieved excellent results with regard to bodyweight and glycemic control as has been described previously [6]. However, both mean HbA1c and weight have increased over the last 2 years.

Table 2 shows mean status for the controls who have switched to a carbohydrate-restricted diet. Three more controls have attempted a change of diet at later dates with varying success.

**Table 2:** Mean weight and HbA1c for the low-fat, high-CHO group (n = 7) at start and after 6–8 months on a high-CHO diet (months 0 and 8), and after cross-over to low-carbohydrate at months 15, 22 and 44.

**Months**	**Weight **(kg)	**HbA1c **(%)
**0. (start High-CHO)**	**99.7 **± 17.7	**7.1 **± 1.2
**8 (start Low-CHO)**	**96.5 **± 19.4	**6.6 **± 0.7
**15**	**89.1 **± 16.0	**5.7 **± 0.8
**22**	**91.9 **± 17.0	**6.0 **± 1.0
**44**	**93.7 **± 17.3	**6.5 **± 0.8

### Diabetes free

Of the total of 10 controls, who have switched diet, 2 persons after a weight reduction of 20 kg each are free of all signs of diabetes after 3 and 2 years respectively i.e. HbA1c below 5.0%, fasting blood glucose below 5.0 mmol/l and free of any blood glucose lowering medication.

### Cardiovascular disease

We have examined medical charts for episodes of cardiovascular disease beginning 3 months after the initiation of the diet therapy.

Among the 16 patients in the low-carbohydrate diet group (41 months observations time) and among the 7 controls that changed from the high-carbohydrate diet to the opposite (33 months observations time) – totalling 23 patients – 2 patients have suffered cardiovascular disease, stroke and heart failure respectively (8.5%. 95% confidence interval (CI 95%): 1.0–28.0). One patient without known cardiac disease has died suddenly. Autopsy showed no sign of coronary thrombosis, myocardial infarction or stroke. The cause of death unknown but assumed to be general atherosclerosis.

As for the 3 controls who switched diet at later dates, there has been no occurrence of cardiovascular disease.

Four patients (80%. CI 95%: 28.3–99.5) among the 5 controls that never attempted any change of diet have suffered several heart infarctions followed by heart failure. Two of them have died from their heart disease (p = 0.025. Fischer Exact).

## Discussion

A summary of the implications of the current work:

1. This year, for the first time, the ADA accepted the value of carbohydrate-restricted diet for weight loss. The text of their guidelines, however, continues previous guidelines in finding fault with such diets and, in fact, does not cite most of the recent publications supporting their use [2]. Other health agencies have similarly insisted on low fat approaches. There is reason to believe that these guidelines are not followed in practice. A perusal of internet diabetes sites suggests that the major dietary emphasis is on carbohydrate control.

2. The major barrier to official acceptance is the stated lack of long term trials although it has never been stated what the features of successes in short term trials suggest that they would not be maintained.

3. The work presented here suggests the importance of funding large scale long term trials as well as the benefits and limited risk in using low carbohydrate diets now.

4. Several studies have shown that low fat diets can be successful but overall, it would be difficult to say they are inherently reliable.

5. In the studies reported here, patients in the two groups had, despite all possible support, failed in achieving an acceptable control of bodyweight and hyperglycemia on traditional low fat diets.

8. An important issue is the fact that some patients do become completely free of disease as soon as they are presented with a low-carbohydrate option. It is unknown what factors make these persons succeed now despite complete failure in the past.

In the low-carbohydrate group bodyweight and HbA1c is still significantly lower than before start. The bodyweight of 7 patients (43%) is still 10% below the initial weight, the original goal of the study. The success rate almost 4 years later is thus 43% as compared to zero in the control group.

Five of 16 patients in the intervention group have had stable bodyweight 38 months after the conclusion of the 6 months study period without any special follow-up. Weight increase has been preceded by an increased intake of carbohydrates in those cases where it has occurred. It is clear that the high-carbohydrate diet followed before the study has been an important, probably the central, contributing cause of their condition.

One rationale for a low-carbohydrate diet is the experimentally observed reduction in hunger [8] Patients generally reported that hunger was absent on the intervention diet and only after increasing dietary carbohydrates did it return.

The intensity of hunger has been reported to be positively correlated to the proportion of carbohydrates in obese men over a 4 week period [9].

We believe that the close follow-up was important. Patients had many questions at each meeting and concerns about the diet that might have hindered adherence were cleared up. In additions individual patients received support from the group.

There is now little evidence for the claim that a fat-reduced diet for weight reduction has any particular value beyond caloric counting [10]. On the other hand, six randomised studies have shown that carbohydrate restriction with ad-libitum energy intake confers a significant benefit with regard to weight loss in obese persons [11-16]. The current study is consistent with these reports and suggests that high-starch, high-carbohydrate diets excessively stimulate appetite and disturb energy balance in patients with the metabolic syndrome and type 2 diabetes [3]. A reduction of carbohydrates normalises the balance, reduces insulin concentrations and favours utilization of stored fat as fuel as well as significantly reducing insulin resistance [3]. Considering the solid evidence for the negative effect of hyperglycemia on diabetes complications as well as cardiovascular disease the present high-carbohydrate dietary advice resulting in unnecessary hyperglycemia and insulin resistance seems difficult to support [17-19] and for diabetes patients, current dietary recommendations seem to be a major part of their problem rather than being part of the solution. Carbohydrate restriction, however, reverses or neutralises all aspects of the metabolic syndrome [20,21].

Summary: A reduced carbohydrate diet is effective in motivated patients and can be recommended for overweight patients with type 2 diabetes. There has been no sign of a negative cardiovascular effect.

## Competing interests

The authors declare that they have no competing interests.

## Authors' contributions

JVN wrote the manuscript and analysed the data. Both authors collected data and gave final approval to the manuscript.
